# Histomorphometric and immunohistochemical evaluation of collagen containing xenogeneic bone blocks used for lateral bone augmentation in staged implant placement

**DOI:** 10.1186/s40729-017-0087-1

**Published:** 2017-06-21

**Authors:** Alberto Ortiz-Vigón, Sergio Martinez-Villa, Iñaki Suarez, Fabio Vignoletti, Mariano Sanz

**Affiliations:** ETEP Research Group, Facultad de Odontología, Universidad Complutense de Madrid, Plaza Ramón y Cajal, 28040 Madrid, Spain

**Keywords:** Clinical trial, Bone regeneration, Alveolar ridge augmentation, Xenogeneic bone substitutes, Heterografts, Histology, Immunohistochemistry, Dental implants

## Abstract

**Background:**

The osteoconductive properties of collagen containing xenogeneic bone blocks (CCXBB) remain unclear. The aim of this prospective single-arm clinical study was to assess the histological outcomes of CCXBB blocks used as bone replacement grafts for lateral bone augmentation procedures.

**Methods:**

In 15 patients with severe horizontal alveolar ridge resorption, lateral augmentation procedures were performed using CCXBB as bone replacement grafts. Twenty-six weeks postoperatively, a re-entry procedure was performed to evaluate the bone width for adequate implant placement and two histological specimens were retrieved from each patient, one being processed for ground sectioning and the other for decalcified paraffin-included sections. In non-decalcified sections, the relative proportions occupied by bone, biomaterials, and connective tissue present in the biopsies were identified. In de-calcified sections, structures and cells positive for osteopontin (OPN), tartrate-resistant acid phosphatase activity (TRAP), osteocalcin (OSC), and alkaline phosphatase (ALP) were assessed.

**Results:**

Soft tissue dehiscence occurred during the follow-up in 5 out of 15 patients (33.3%). The mean crest width at baseline was 2.78 mm (SD 0.57) and the mean crest width at re-entry was 6.90 mm (SD 1.22), with a mean ridge width increase of 4.12 mm (SD 1.32). Twenty-six bone biopsies were obtained from 13 patients. Histomorphometric analysis showed a mean of 26.90% (SD 12.21) of mineralized vital bone (MVB), 21.37% (SD 7.36) of residual CCXBB, 47.13% (SD 19.15) of non-mineralized tissue, and 0.92% of DBBM. The immunohistochemical analysis revealed a large number of OPN-positive cells 8.12% (SD 4.73), a lower proportion of TRAP positive multinuclear cells 5.09% (SD 4.91), OSC-positive cells 4.09% (SD 4.34), and a limited amount of ALP positive cells 1.63% (SD 2).

**Conclusions:**

CCXBB achieved significant horizontal crestal width allowing for staged implant placement in most of the patients. In light of the histological outcomes and implant failures, special attention must be placed to prevent soft tissue dehiscence when CCXBB is used in severe atrophic alveolar crests.

## Background

Different techniques and grafting materials have been used for the horizontal reconstruction of deficient alveolar processes before implant placement, resulting in different degrees of predictability and clinical outcomes [1]. Among the grafting materials, particulated xenogeneic materials have been extensively studied in both experimental and clinical studies and when combined with porcine-derived natural collagen membranes have resulted in predictable clinical and histological outcomes [2].This combined treatment has shown to be safe and efficacious in horizontal ridge augmentations resulting in regenerated bone with similar implant survival rates when compared to implants placed in pristine bone and demonstrating a low degree of morbidity and a low rate of post-operative complications [3, 4]. However, in clinical situations with severe bone resorption of the alveolar process, which results in non-self-containing bone defects, the use of particulate bone replacement grafts with its inherent weak volumetric stability may limit the predictability of the regenerative therapy [5, 6]. In these cases, dental implants are usually placed staged to the lateral bone augmentation procedure and autogenous bone blocks have been the state of the art procedure, resulting in superior results in mean horizontal bone gains when compared with guided bone regeneration with particulate bone grafts [4, 7, 8]. The use of bone blocks, however, has been associated with increased surgical time, morbidity [9, 10], and a higher frequency of post-operative complications [11, 12]. Moreover, the availability for harvesting intraoral bone blocks is limited [13, 14] and these autologous bone grafts may suffer a high degree of bone resorption during healing [15].

To overcome these limitations, the use of xenogeneic bone grafts as an alternative to autogenous bone blocks has been proposed [16]. Recently, a new equine-derived collagen containing xenogeneic bone block (CCXBB) was evaluated in preclinical studies [17, 18], demonstrating to be safe and adequate for ridge augmentation and better graft integration when compared to other xenogeneic bone blocks. Its performance in humans has been recently tested on 10 patients where these xenogeneic bone blocks were placed in single-tooth alveolar bone defects [19]. Clinically, a mean horizontal gains of 3.88 ± 1.75 mm was reported and the histological outcomes resulted in a homogeneous new bone formation within the CCXBB. These results were concordant with a recent histological study also reporting that equine bone grafts were biocompatible and underwent advanced remodelling at the time of implant placement [20]. Although this preliminary evidence on the performance and histological behaviour of equine bone blocks seems promising, there is still limited information when used in staged horizontal bone augmentation of large osseous defects. It was, therefore, the aim of this prospective study to evaluate the histological outcomes of CCXBB blocks used for lateral bone augmentation in large alveolar horizontal defects of at least two adjacent missing teeth.

## Methods

### Study design

The present manuscript reports the histological outcomes of a prospective single arm study evaluating the safety and clinical performance of CCXBB blocks when used as replacement bone grafts for lateral bone augmentation prior to staged implant placement. The results of the clinical and radiographic outcomes have been reported in a previous publication [21]. For correlation of the histological with the clinical outcome, respective data of the previous publication have been inserted.

### Patient sample

Adults (≥18 years of age) were screened on the bases of having single or multiple teeth absences and a severe horizontal collapse of the alveolar ridge in need of one or more implants for implant supported fixed prosthetic rehabilitation.

Patients were selected on the bases of fulfillment of the following inclusion and exclusion criteria:

- Written informed consent
- Insufficient bone ridge width (<4 mm) for implant placement measured on a cone beam computed tomography (CBCT)
- Sufficient bone height for implant placement
- Healthy oral mucosa and ≥3 mm of attached keratinized mucosa

Patients were excluded if they had any of these conditions:

- General contraindications for dental and/or surgical treatments

Inflammatory and autoimmune disease of the oral cavity

- Allergy to collagen
- Diabetes
- History of myeloma, respiratory tract cancer, breast cancer, prostate cancer or kidney cancer requiring chemotherapy or radiotherapy within the past 5 years

Concurrent or previous radiotherapy of head area

- Concurrent or previous immunosuppressant, bisphosphonate, or high-dose corticosteroid therapy
- Smokers
- Pregnant or lactating women
- Women of child bearing age, who are not using a highly effective method of birth control
- Participation in an investigational device, drug, or biologics study within the last 24 weeks prior to the study start

Before final inclusion, patients received meticulous verbal and written descriptions of the interventions and conditions and were requested to sign an informed consent form (directive 95/46/EC on data protection, in accordance with current legal provisions by the European Community).

### Experimental product information

CCXBB (Bio-Graft® Geistlich Pharma) is a bone substitute material in a natural block form. The dimensions of the Bio-Graft block are 10 mm in height, 10 mm in length and 5 mm in width. It consists of a natural cancellous bone structure of hydroxyapatite and endogenous collagen type I and III, equine origin and is a class III medical device according to the Medical Device Directive 93/42 EECs’ definition (rule 8 implantable, resorbable device) and 17 (animal origin) in annex lX CE certificate G7 11 04 39446 050 for Geistlich Bio-Graft® was issued in June 2011.

The manufacture of Geistlich Bio-Graft is according to a standardized, controlled process and good manufacturing practices (GMPs). Each batch is manufactured and documented according to standard operating procedures, and the entire process has been validated.

### Outcomes variables

**Fig. 1:**
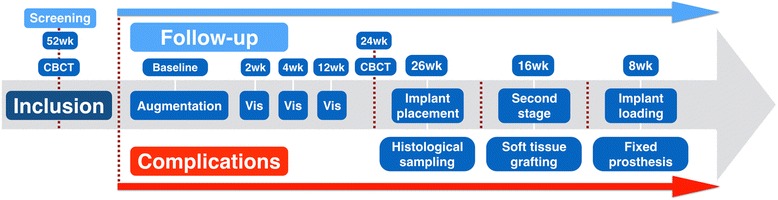
Study chart and follow-up visits

The study design and follow-up visits have been summarized in Fig. 1. The primary outcome of this study was to assess the performance of the CCXBB by measuring the final crestal ridge width after 6 months of healing and evaluating its appropriateness for implant placement and the occurrence of adverse effects during healing.

Furthermore, the histological outcomes of this xenogeneic bone replacement graft were evaluated by harvesting a core biopsy of the regenerated area immediately before implant placement (after 6 months of healing), as well as the implant survival of those implants placed in the regenerated bone.

### Surgical procedure and clinical measurements

**Fig. 2:**
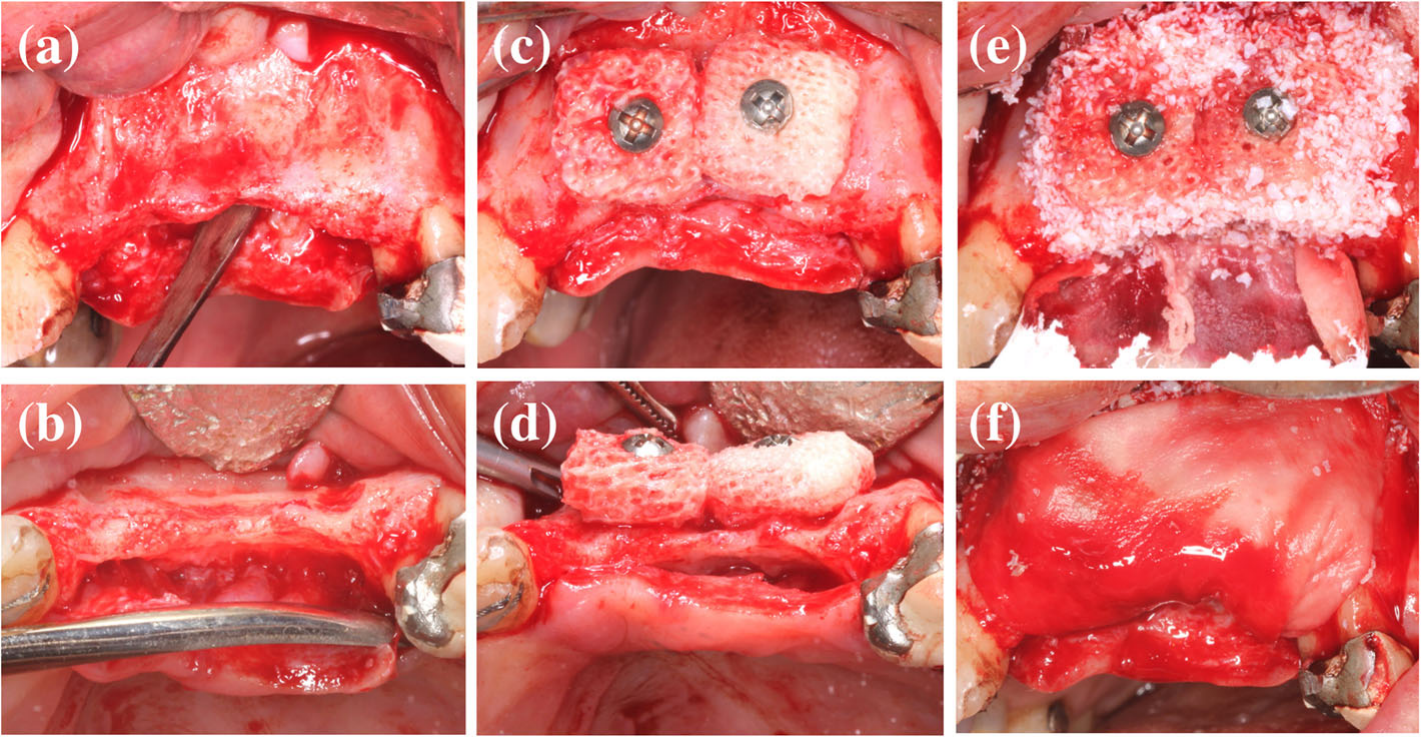
Lateral bone augmentation of the alveolar crest (**a**) atrophic ridge. **b** Perforations and adaptation of the cortical layer. **c** Shaping, pre-wetting and fixation of CCXBB with titanium screws. **d** Horizontal contour and peripheral gap between CCXBB and bone layer. **e** Outlying DBBM filling. **f** CM stabilized with pins

The surgical placement of the CCXBB blocks and the clinical evaluation has been described in detail in a previous publication [21]. In brief, severe alveolar horizontal bone deficiencies were isolated after rising full-thickness mucoperiosteal flaps. Once the horizontal width of the alveolar crest was measured 2 mm below the crest with a bone calliper bone blocks were shaped, pre-drilled and pre-hydrated for 5 min with sterile physiological saline before placement and were fixed with titanium osteosynthesis screws allowing for a stable contact between the block graft and the underlying bone. The spaces between the bone block and the surrounding bone were filled with DBBM particles (Geistlich Bio-Oss®, Geistlich Pharma AG, Wolhusen, Switzerland) and covered with a native collagen membrane (CM) (Geistlich Bio-Gide®, Geistlich Pharma AG, Wolhusen, Switzerland) fixed to the underlying bone with titanium tacks (FRIOS Fixation-Set®, SYMBIOS, Mainz, Germany). The muco-periosteal flaps were then coronally advanced and sutured achieving a tension-free primary closure (Fig. 2).

### Bone biopsies harvesting procedure

Twenty-six weeks after the regenerative procedure the patient returned for the re-entry intervention for placement of dental implants. After raising full-thickness flaps, the augmented area was exposed and horizontal crestal width measurements were performed. Then, the surgeon evaluated the bone availability and if implant placement was considered possible, a core bone biopsy was harvested with the use of a trephine, replacing the first drill of the implant bed preparation (2 mm diameter and 10 mm length, Hager and Meisinger® Neuss, Germany).

**Fig. 3:**
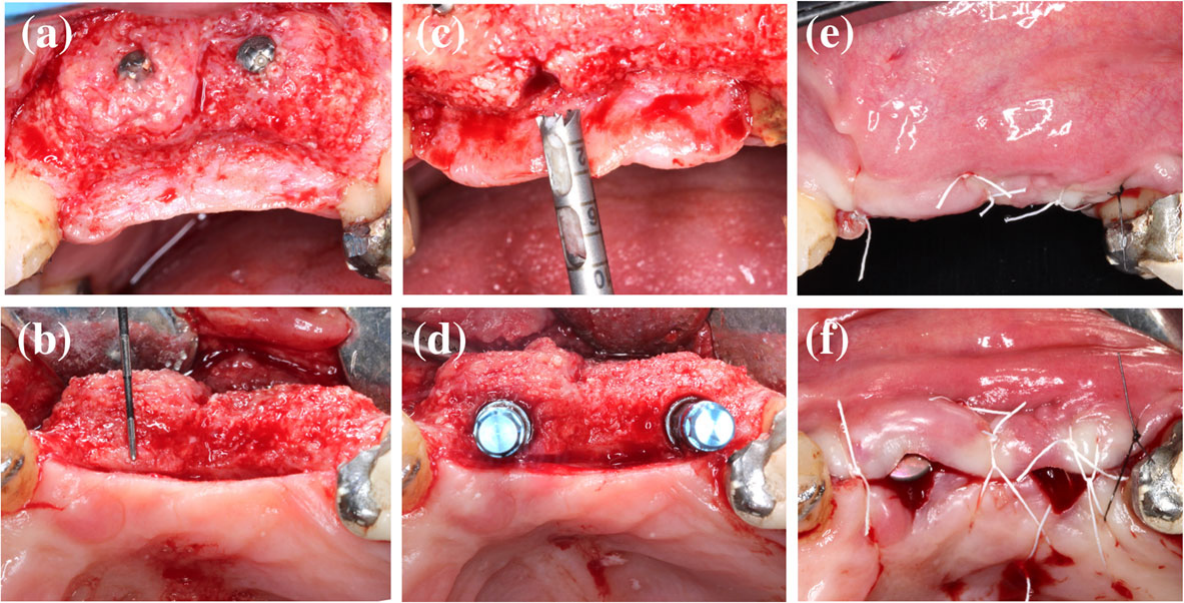
Re-entry procedure of patient in Fig. 1. **a** Buccal aspect of the augmented region. **b** Horizontal bone augmentation. **c** Screws and pins removal and bone trephine sampling. **d** Implants placement and buccal bone width from the implant shoulder. **e** Primary flap closure. **f** Implants submerged healing

The retrieved trephine containing the bone biopsy was irrigated with saline to remove the blood and was introduced in a tube containing 10% formalin solution, which was coded and stored until processing. Commercially available titanium dental implants were inserted in accordance with manufacturer guidelines and after 8 weeks of healing, fixed screwed-retained prosthetic restorations were placed (Fig. 3).

### Histological processing

One biopsy per patient was processed for ground sectioning according to the method described by Donath and Breuner (1982). In brief, the specimens including the trephines were fixed in neutral-buffered formalin, stored in compartment biopsy cassettes, and appropriately coded for identification. Once fixed, the blocks containing the trephines were dissected, dehydrated with ascending alcohol grades and embedded in a light-curing resin (Technovit 7200 VLC; Heraeus-Kulzer, Wehrheim, Germany). At least two longitudinal sections of each core biopsy were grounded and reduced to a thickness of approximately 40 microns using Exakt cutting and grinding equipment (Exakt Apparatebau, Norderstedt, Germany). All the sections were stained using the Levai-Laczkó technique [22].

The second biopsies were processed for decalcification, included in paraffin, stained with hematoxyline-eosine (H-E) and further processed for immune-histochemical analysis. The biopsies were fixed overnight in 4% neutral buffered formalin. Decalcification was achieved by immersing the specimens in 1 mM EDTA solution and then embedded in paraffin following standard procedures. Semi-thin sections of 4-μm-thick were obtained and stained with hematoxyline-eosine (H-E).

For the immunohistochemical analysis, the semi-thin sections were incubated over night with primary antibodies at 4 °C (Santa Cruz Biotechnology Inc., Santa Cruz, Calif., USA). The antibody dilutions used were alkaline phosphatase (ALP) 1:100, osteopontin (OPN) 1:100, osteocalcin (OSC) 1:100, and tatrate resistant acid phosphatase (TRAP) 1:100.

### Histological analysis

#### Qualitative analysis

**Fig. 4:**
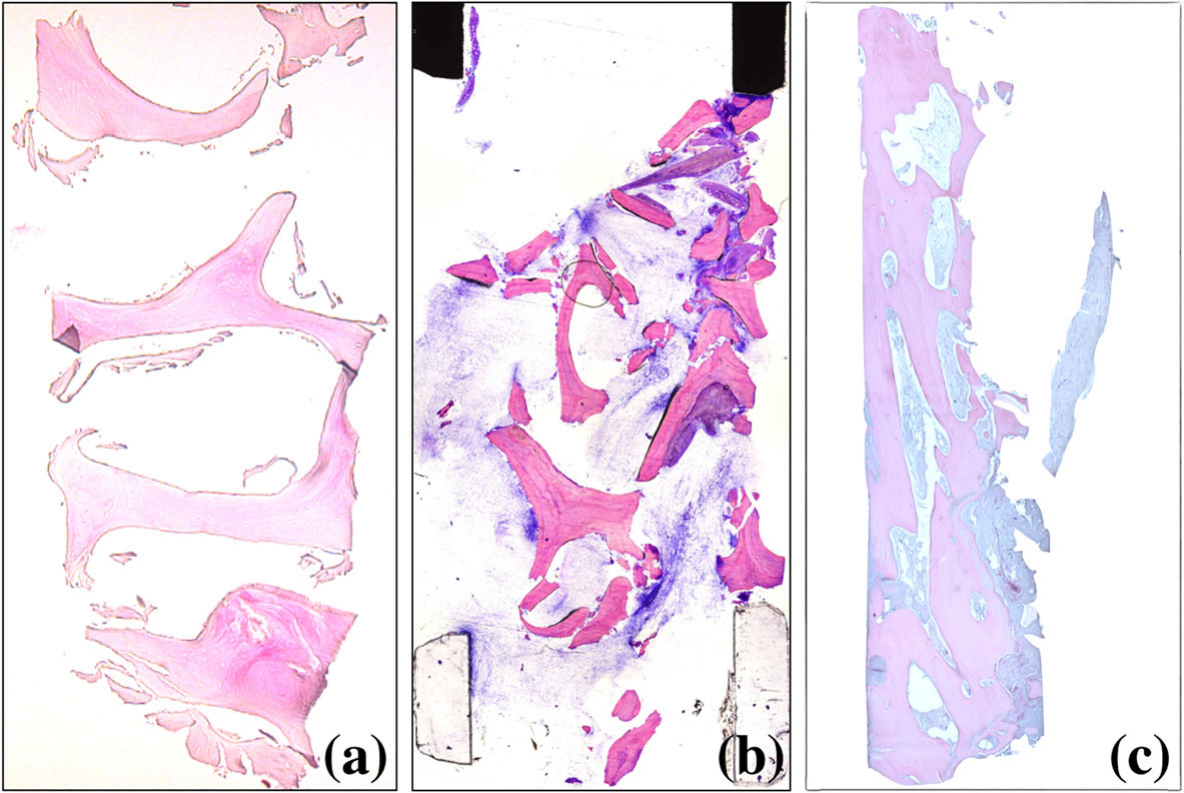
Histological samples. **a** CCXBB control without implantation. **b** Histologic samples with acute inflammatory infiltration. **c** Histologic sample with limited remaining CCXBB and large bone ingrowth

The obtained semi-thin sections were evaluated with a motorized (Märzhäuser, Wetzlar-Steindorf, Germany) light microscope connected to a digital camera and a PC-based image-capture system (BX51, DP71, Olympus Corporation, Tokyo, Japan). Photographs were obtained at ×5 and ×20 magnifications (Fig. 4).

#### Histomorphometric analysis

**Fig. 5:**
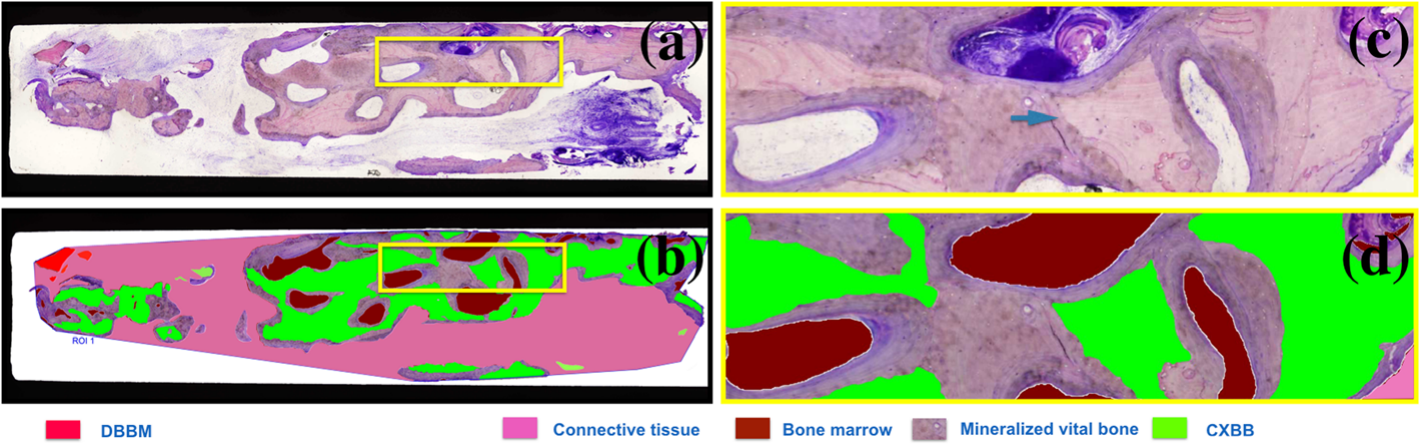
Histomorphometric analysis of the same sample. **a** Ground section stained with Levai-Laczkó. **b** Tissue identification of the ROI. **c** Closer view **a** *arrow* pointing a cement line between new mineralized bone and CCXBB. **d** Closer view of **b**

From the obtained images, areas within the biopsies occupied by bone, biomaterial and connective tissue were identified using a pen computer (Cintiq companion, Wacom, Düsseldorf, Germany), coloured (Photoshop, Adobe, San José, CA, USA) and digitally measured using an automated image-analysis system (CellSens, Olympus Corporation) (Fig. 5).

#### Immunohistochemical analysis

**Fig. 6:**
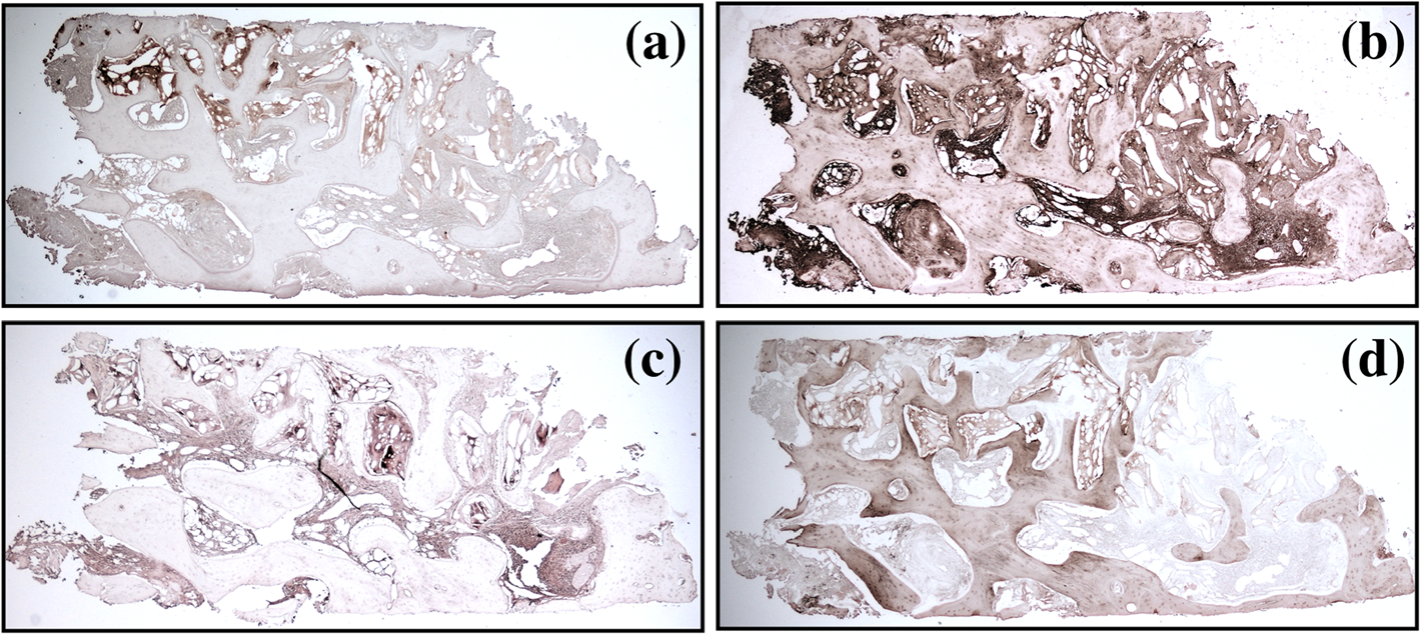
Immunohistochemical analysis of slices from the same sample with four different markers. **a** TRAP. **b** OPN. **c** ALP. **d** OSC

The obtained histological sections were observed in a light microscope using 5x magnification. In the centre of each trephine biopsy, a rectangular region of interest (ROI) with a size of 30,000,000 to 32,000,000 pixels was defined and standardized photographs were obtained. The intensity of the antibody staining in the images was analysed using the software ImageJ, which by evaluating the antibody staining intensity in the area of interest allows for assessing quantitatively the specific marker (*ImageJ®*, *IHC Profiler plugin)*. With this tool, the specimens were categorized into four groups: high positive (HP), positive (P), low positive (LP), and negative (N). To reduce false positives, only the HP and P values were considered for evaluating the percentage of positiveness for each immunohistochemical marker (Fig. 6).

### Statistical analysis

Data were entered into an Excel (Microsoft Office 2011) database and proofed for entry errors. The software package (IBM SPSS Statistics 21.0; IBM Corporation, Armonk, NY, USA) was used for the analysis. A subject level analysis was performed for each outcome measurement reporting data as mean values, standard deviations, medians, 95% confidence intervals (CI), and frequencies. Shapiro–Wilk goodness-of-fit tests were used to assess the normality and distribution of data. Descriptive analysis of the histological and immunohistochemical outcomes was carried out by reporting means and standard deviations and comparisons between these histological outcomes between patients with subsequent implant loss versus patients with successful implant outcomes were evaluated using the paired sample *t* test or *U* Mann-Whitney if the distributions were non-normalized. Results were considered statistically significant at *p* < 0.05.

**Fig. 7:**
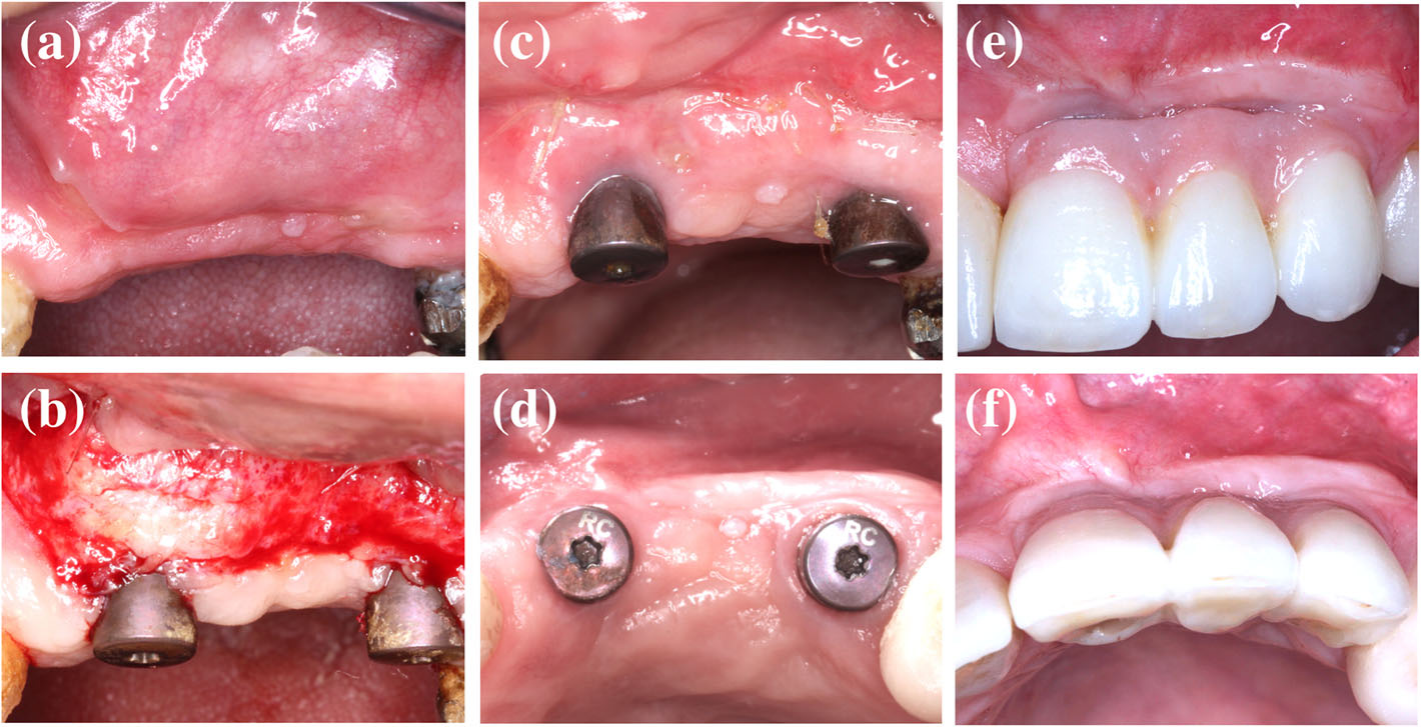
Second stage surgery of patient in Fig. 1. **a** Vestibular depth reduction after augmentation and implant placement. **b** Partial thickness and apical repositioned flap. **c** CMX healing and soft tissue dehiscence with CCXBB exposure. **d** Dehiscence healing after re-contouring and buccal emergency profile. **e** Buccal aspect of the final restoration. **f** Buccal ridge contour

## Results

Twenty-eight CCXBB blocks were placed in 15 patients that fulfilled the selection criteria (12 women and 3 men) with a mean age of 54.5 (SD 8.34).

### Clinical results

The detailed clinical and radiographical outcomes have been reported previously [21]. In brief, one patient experienced pain and soft tissue dehiscence leading to removal of the graft material 3 days after the regenerative procedure. Another patient refused to proceed to implant placement after suffering an early dehiscence also leading to a complete removal of the graft. From the remaining 13 patients completing the study, the alveolar ridge width augmented from a mean 2.78 mm (SD 0.55) at baseline to 6.90 mm (SD 1.22) at re-entry, resulting in a statistical significant mean alveolar crest width gain of 4.12 mm (SD 1.32) Sixteen weeks after implant placement a second stage procedure and a soft tissue augmentation was performed (Fig. 7).

**Fig. 8:**
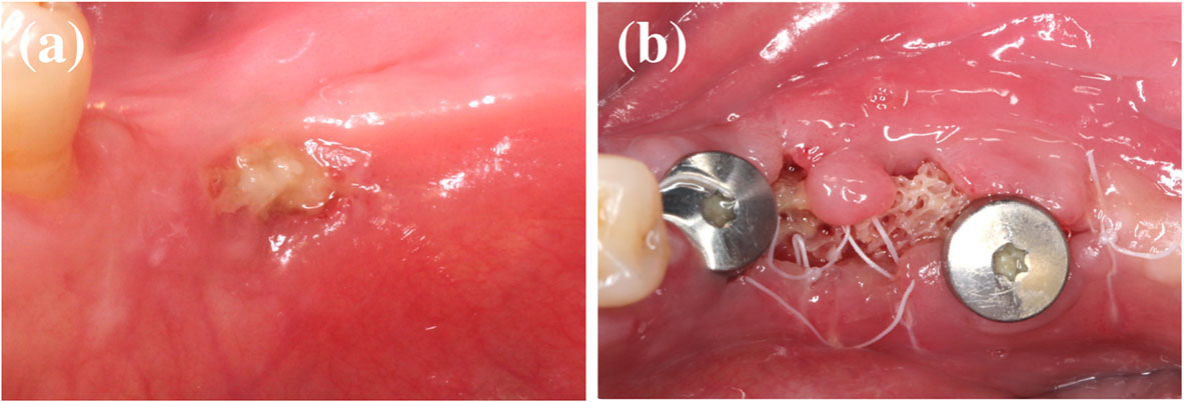
Soft tissue dehiscence (**a**) CCXBB exposure 15 weeks after bone augmentation, the dehiscence healed 2 weeks later after reducing the graft exposure (**b**) after soft tissue augmentation and abutment connection leading to the loss of the mesial implant. After partial removal of the bone graft and place a connective tissue graft the area healed properly and a month later it was possible to replace the implant

Although soft tissue dehiscence, with different degrees of graft exposure, occurred at different time points in 5 out of 15 patients (33.3%) (Fig. 8), 24 implants were placed in 13 patients. Table 1 depicts the data on survival rates at the time of loading. Three implants were lost in three patients at the time of abutment connection, and one patient presenting very narrow ridge at baseline (<2 mm) lost all the implants. Nevertheless, all implants could be replaced without additional grafting procedure.

**Table 1:** Clinical and histomorphometry assessments (i.e., dehiscences, mineralized bone, CCXBB, bone marrow, connective tissue, and implant lost)

Patient	Soft tissue dehiscence	Mineralized bone (%)	CCXBB (%)	Bone marrow (%)	Connective tissue (%)	Implant lost
1	No	22.56	25.26	15,443	36.03	No
2	*Yes*	0	28.49	0.000	71.50	*Yes*
3	No	30.39	21.10	14,248	33.24	No
4	No	31.59	21.79	13,982	32.62	No
5	No	41.44	11.90	17,259	25.88	*Yes*
6	*Yes*	39.41	5.88	54,461	0	No
7	No	24.31	13.21	32,813	26.84	No
8	*Yes*	26.19	21.09	52,714	0	No
9	No	23.39	30.18	39,455	6.96	No
10	No	17.73	15.79	26,590	39.88	*Yes*
11	*Yes*	12.95	27.09	10,104	48.83	*Yes*
12	No	37.45	21.37	35,035	3.89	No
13	No	42.31	28.35	29,129	0	No
%	Yes: 30.7 No: 69.2	26.90	20.89	26.24	25.05	Yes: 30.7 No: 69.2
Median		26.19	21.37	26.59	26.84	
SD		12.21	7.35	16.43	22.07	
IR		18.28	13.22	23.13	36.01	
95% CI		19.52;34.28	16.44;25.33	16.31;36.18	11.71;38.39	

*Abbreviations*: *CCXBB* Collagen containing xenogenic bovine bone, *SD* Standard deviation, *IR* Interquartile range, *CI* Confidence interval

### Histological observations

Histological biopsies from 13 patients were harvested and processed for histological analysis. The histomorphology of the healed CCXBB bone grafts evidenced in most of the samples newly formed mineralized vital woven bone, as well as residual graft material, bone marrow, and non-mineralized connective tissue (Fig. 5). Residual CCXBB appeared to be integrated with the new bone, which had grown within the graft trabeculae. CCXBB and DBBM were identified only by the presence of empty lacunae and cement lines separating the graft from the parent bone (Fig. 5c). In four of the specimens analyzed, minimal or no signs of new bone formation were appreciated, showing an inflammatory infiltrate with neutrophils and macrophages associated with tissue destruction (Fig. 4b).

### Histomorphometrical results

The results from the histomorphometric measurements are depicted in Table 2. Bone biopsies were composed by 21.37% (SD 7.36) of residual CCXBB, 26.90% (SD 12.21) of mineralized vital bone (MVB), 47.13% (SD 19.15) of non-mineralized tissue and 0.92% of DBBM (Fig. 5b). Biopsies from patients who lost their implants had a statistical significant lower amount of MVB (*p* = 0.01^u^) and a statistical significant larger proportion of connective tissue (*p* = 0.02^t^) (Table 4). Furthermore, although no statistically significant correlation was observed between presence of soft tissue dehiscence and specific histomorphological outcomes, a tendency towards a low amount of new bone was observed in the specimens from patients where the bone graft had been exposed (*p* = 0.06).

**Table 2:** Quantitative histological analysis

Tissue type	Mean	Standard deviation	Median	CI 95%
Mineralized bone	2.8 × 10^6^	1.2 × 10^6^	3.1 × 10^6^	2.0 × 10^6^–3.5 × 10^6^
Connective tissue	2.7 × 10^6^	2.7 × 10^6^	3.1 × 10^6^	1.0 × 10^6^–4.4 × 10^6^
Bone Marrow	2.8 × 10^6^	2.0 × 10^6^	2.2 × 10^6^	1.6 × 10^6^–4.1 × 10^6^
CCXBB	2.3 × 10^6^	1.2 × 10^6^	2.4 × 10^6^	1.6 × 10^6^–3.1 × 10^6^
DBBM	8.9 × 10^4^	1.2 × 10^5^	2.0 × 10^4^	1.6 × 10^4^–1.6 × 10^5^

### Immunohistochemical results

Results from the immune-histochemical analysis are presented in Table 3. A large number of OPN-positive cells (mesenchymal, hematopoietic cells and osteoblast) were observed in most of the tissue samples (Fig. 6a). Similarly, TRAP positive multinuclear cells (osteoclasts) were also observed mainly in contact with the residual CCXBB (Fig. 6b). More limited amounts of OSC-positive cells (mature osteoclast) were observed (Fig. 6c) whereas ALP-positive cells (osteoblast) were mainly detected on the surface of the newly formed woven bone and in proximity of vascular units (Fig. 6d). The newly formed bone in close contact with the CCXBB remnants showed signs of modelling and remodelling. When the correlation between the immunohistochemical results and implant loss was investigated, a statistically significant correlation between implant loss and number of OSC positive cells was observed (2 versus 8.78% *p* = 0.02^u^) (Table 4).

**Table 3:** Immunohistochemical markers proportions (i.e., TRAP, OPN, ALP, and OSC)

Patient	TRAP (%)	OPN (%)	ALP (%)	OSC (%)
1	12.36	4.86	3.73	5.79
2	11.68	14.81	0.44	11.17
3	11.01	13.01	0.16	0.72
4	2.05	8.60	4.49	0.95
5	1.81	15.71	0.34	7.63
6	3.21	11.38	1.67	2.81
7	0.22	2.92	0.515	2.92.
8	0.97	9.63	3.95	4.30
9	0.92	5.42	0.02	0.05
10	12.79	4.58	0.01	13.51
11	4.01	10.07	0.18	2.82
12	1.11	2.77	0.22	0.13
13	4.03	1.83	5.45	0.35
Mean	5.09	8.12	1.63	4.09
SD	4.91	4.73	2	4.34
95% CI	2.12;8.06	5.26;10.98	0.41;2.84	1.46;6.71

*Abbreviations*: *TRAP* Tartrate-resistant acid phosphatase, *OPN* Osteopontin, *ALP* alkaline phosphatase, *OSC* Osteocalcine

**Table 4:** Implant loss and tissue characteristics

Differentiated tissues	Implant lost (Yes/no)	Mean	SD	Percentage	SD (%)	Significance (*p* < 0.05)
Mineralized bone	No	*3.5 × *10* ^*6*^	5.1 × 10^5^	*30.84*	7.39	*p = 0.01* ^*u*^
	Yes	*1.3* × *10* ^*6*^	1.2 × 10^6^	18.84	17.31	
Connective tissue	No	1.8 × 10^6^	1.9 × 10^6^	*15.51	16.15	*p = 0.01* ^*t*^
	Yes	4.6 × 10^6^	3.7 × 10^6^	*46.52*	19.14	
Bone marrow	No	*3.7 × *10* ^*6*^	1.9 × 10^6^	*31.91	15.49	*p = 0.02* ^*t*^
	Yes	*1.0* × *10* ^*6*^	9.2 × 10^5^	*13.48*	11.24	
CCXBB	No	2.5 × 10^6^	1.2 × 10^6^	20.92	7.46	*p* = 0.98^t^
	Yes	2.0 × 10^6^	1.4 × 10^6^	20.82	8.22	
TRAP	No	-	-	3.99	4.53	*p* = 0.24^u^
	Yes			7.57	5.47	
OPN	No	-	-	6.71	4.06	*p* = 0.11^t^
	Yes			11.29	5.11	
ALP	No	-	-	2.24	2.15	*p* = 0.09^u^
	Yes			0.24	0.18	
OSC	No	-	-	*2	2.05	*p = 0.02 ^*u*^
	Yes			*8.78*	4.65	

*Abbreviations*: *t* T de student, *u* Mann–Whitney, *Statistically significant

## Discussion

The purpose of this investigation was to evaluate histologically and immunohistochemically the behavior of CCXBB blocks when used for staged lateral bone augmentation in severe human horizontal residual bone defects. Six months after the regenerative intervention using the CCXBB blocks, the mean increase in bone width was 4.12 mm and hence, this outcome allowed for the placement of dental implants in 11 out of 15 patients (73.3%). These results were concordant with the reported weighted mean width increases (3.90 mm (SD 0.38)) from a recent systematic review evaluating intraoral autogenous bone blocks [4]. These results were also similar to those reported with the use of allogeneic bone blocks (4.50 mm (SD 1.3)) [23] or with those from a pilot study using the same CCXBB xenogeneic bone blocks for the staged regeneration of single tooth bone defects (Schwarz, et al. 2016). In this study, in eight patients, the mean crestal width gain was 3.88 mm (SD 1.75) and implant placement was feasible in eight out of ten (80%) patients at re-entry [19].

To the best of our knowledge, the present investigation represents the first study reporting histomorphometric and immunohistochemical outcomes of the use of CCXBB for regenerating atrophic alveolar bone in humans. The healing after 26 weeks was characterized in most of the samples by newly formed mineralized vital bone containing viable osteocytes, as well as bone marrow and non-mineralized connective tissue. This new bone was observed in intimate contact with the residual CCXBB. The percentages of mineralized vital bone, bone marrow, and connective tissue were 26.9, 26.2 and 25.1%, respectively. Similar proportions have been reported with the use of allogeneic blocks [23, 24]. With autogenous bone blocks the relative tissue composition attained was 25.1% of vital bone, 18.1% of connective tissue, and 56.7% of necrotic bone [25]. Similarly, [26] reported 57.75% of non-vital bone when using autogenous bone blocks. In the present investigation CCXBB was present in 21.4% of the samples after 26 weeks of healing, what is in agreement with previous studies reporting histological outcomes of other xenogeneic bone replacement grafts placed for the regeneration of extraction sockets [27, 28]. In this indication, the percentage of residual graft was 39.8 and 33.4%, respectively.

When correlating the clinical results and the histological outcomes, there was a positive association between the presence of soft tissue dehiscence with CCXBB exposure and a diminished amount of new mineralized bone (*p* = 0.06). This lower amount of new bone within the xenogeneic graft suggests a lack of full graft integration and diminished vascular supply, what may have caused the soft tissues dehiscence. Similarly, the biopsies from patients who lost their implants had a statistical significant lower amount of MVB and a statistical significant larger proportion of connective tissue, what suggests that there is a direct relationship between the primary healing of the bone replacement graft, its integration with native bone and its healing to provide a biological base for dental implants to osseointegrate. These results corroborate the importance of minimal trauma during surgery, establishment of primary implant stability and avoidance of infection and micromotion during healing as key prerequisites for achieving dental implant osseointegration [29, 30]. In fact, the high incidence of early implant loss (29.2%) reported in this clinical study, is clearly higher when compared with epidemiological data from Sweden reporting early implant loss in 4.4% of patients and 1.4% of implants [31]. The delayed bone proliferative phase has also been described associated with other bone replacement grafts for bone regeneration [32] and with demineralized bovine bone mineral (DBBM) in the healing of fresh extraction sockets [33].

A high incidence of soft tissue dehiscence and implant failures has been reported in patients receiving fresh frozen allogeneic bone grafts for reconstructing severe alveolar atrophies (36.8% incidence of dehiscence and 31.5% incidence of implant loss) [34] and 21% of implant loss [35], respectively. With the use of a different equine bone block, a previous publication reported total removal of the graft in 50% of the patients and in 20% of them the implants failed [36]. The high incidence of soft tissue dehiscence occurring in this clinical study may also be explained by the extreme narrow crestal defects (mean crestal width of 2.78 mm) multiple teeth absence and non-containing defects, what needed in most of the cases to use more than one block graft. In fact, there was a positive correlation between the number of blocks used and the incidence of soft tissue dehiscences. The use of large grafts or more than one graft may have hindered an appropriate blood supply or colonization of the graft material with bone-forming cells [37].

The immune-histochemical results reported expression of osteopontin mainly at the border between mineralized vital bone (MVB) with CCXBB, what coincides with findings from previous reports [38–40]. Alkaline phosphatase (ALP) is considered as an early osteoblast differentiation marker [41]. ALP-positive cells were detectable, in all specimens on the periphery of MVB, associated to areas of new bone formation. These observations were also reported on a clinical study on guided bone regeneration (GBR) [41], as well as through the evaluation of the healing of particulate xenogeneic bone grafts (DBBM) [28]. Experimental research using immune-histochemical analysis for comparing early bone remodelling between autografts and allografts has reported comparable behavior for osteoprotegerin (OPG), alkaline phosphatase (ALP), collagen 1 (COLI), osteopontin (OPN), and osteocalcin (OSC), although an increased activity of tartrate-resistant acid phosphatase (TRAP) was seen in allogenic bone grafts [42]. In this investigation TRAP, which is a specific enzyme present in large quantities at the osteoclasts edge expressing bone resorption, was present in high proportions in all the analysed samples. On the other hand, OSC (bone matrix protein), predominantly synthesized by osteoblasts, has a fundamental role in bone formation (mineralization) and resorption [43]. Experimental studies have demonstrated the role of OSC during the early healing phases of osseointegration of dental implants [44]. In the present investigation, a statistical significant correlation between higher levels of OSC and implant loss was found. This association could be explained by a greater activity of bone modelling in these situations of deficient mineralization [45].

This prospective single-arm study has clear limitations to evaluate the efficacy of this bone regenerative intervention, since there is not a control group [46]. However, this investigation has shown excellent clinical performance and histological outcomes when CCXBB were used for lateral bone augmentation and when their integration occurred without soft tissue dehiscence.

## Conclusions

Within the limitations of this clinical study, we may conclude that the use of CCXBB in combination with DBBM particles and a native bilayer collagen membrane for staged lateral bone augmentation in severe atrophic alveolar crests achieved significant horizontal crestal width allowing for staged implant placement in most of the patients. Histological analysis and implant survival records indicate that special attention must be paid to prevent soft tissue dehiscence.

## Abbreviations

ALP: Alkaline phosphatase
CBCT: Cone beam computed tomography
CCXBB: Collagen containing xenogeneic bone block
CM: Native collagen membrane
DBBM: Deproteinized bovine bone mineral
ETEP: Etiology and Therapy of Periodontal Diseases
OPN: Osteopontin
OSC: Osteocalcine
TRAP: Tartrate-resistant acid phosphatase
